# Does Post-COVID-19 Erectile Dysfunction Improve over Time?

**DOI:** 10.3390/jcm12031241

**Published:** 2023-02-03

**Authors:** Alper Gök, Mehmet Altan, Ahmet Emin Doğan, Aşır Eraslan, Fahrettin Şamil Uysal, Ufuk Öztürk, Ardan Muammer Saguner, Muhammet Abdurrahim İmamoğlu

**Affiliations:** 1 Department of Urology, Faculty of Medicine, University of Health Sciences, Ankara 34668, Turkey; 2 Department of Urology, Etlik City Hospital, Ankara 06170, Turkey; 3 Department of Urology, Diskapi Education and Research Hospital, Ankara 06110, Turkey; 4 Department of Urology, Çubuk State Hospital, Ankara 06760, Turkey; 5 Department of Cardiology, University Heart Center, University Hospital Zurich, 8091 Zurich, Switzerland

**Keywords:** coronavirus, COVID-19, erectile dysfunction, healthcare professional

## Abstract

Background: Some studies have shown that there may be an increase in the frequency of erectile dysfunction after COVID-19. However, no long-term study has investigated whether this is permanent or temporary. In this study, we aimed to examine whether there was an increase in the frequency of erectile dysfunction among individuals with a history of COVID-19, and, if there was, whether their condition improved over time. Materials and methods: In this study, a total of 125 healthy male healthcare workers, 95 with and 30 without a history of COVID-19, were evaluated in terms of erectile function. Four study groups were formed. The first three groups consisted of individuals with a history of COVID-19 confirmed by the polymerase chain reaction (PCR) test at different times, who recovered from the disease (time elapsed since COVID-19 positivity: <6 months for Group 1, 6 to 12 months for Group 2, and >12 months for Group 3). The individuals in Group 4 did not have a history of COVID-19 diagnosis. In order to evaluate the erectile function of the participants, they were asked to complete the five-item International Index of Erectile Function questionnaire (IIEF-5). Then, statistical analyses were performed to evaluate whether there was a difference between the groups in terms of the IIEF-5 scores. Results: There was a statistically significant difference between the groups in terms of the IIEF-5 scores (*p <* 0.001), and this difference was determined to be caused by the significantly higher IIEF-5 scores of Groups 3 and 4 compared to Group 1 (*p =* 0.004 and *p <* 0.001, respectively). In addition, the IIEF-5 score of Group 4 was statistically significantly higher than that of Group 2 (*p <* 0.001). However, the IIEF-5 scores did not statistically significantly differ between Groups 1 and 2, Groups 2 and 3, and Groups 3 and 4 (*p* > 0.999, *p =* 0.204, and *p =* 0.592, respectively). Conclusion: There may be deterioration in erectile function after COVID-19; however, this tends to improve over time, especially from the first year after active infection. Given that vascular, hormonal, and/or psychogenic factors may lead to the development of erectile dysfunction after COVID-19, we consider that in order to easily manage this process, it is important to determine the underlying cause, initiate appropriate treatment, and inform couples that this situation can be temporary.

## 1. Introduction

Coronavirus disease 2019 (COVID-19) caused by severe acute respiratory syndrome coronavirus 2 (SARS-CoV-2), emerged in Wuhan, China in late December 2019 and rapidly became a global health issue, being declared a pandemic on 11 March 2020 [1]. According to the report of the World Health Organization, as of 11 December 2022, the global number of people infected with SARS-CoV-2 was approximately 645 million, while the number of deaths was reported to be 6.6 million [2]. COVID-19, initially considered to only cause upper respiratory tract infection, is now known to affect many tissues and organs [3]. The virus can enter cells by binding to the angiotensin-converting enzyme 2 (ACE-2) receptors in the host through the spike proteins on its surface [4]. Before this binding occurs, spike proteins must be activated by host cellular transmembrane serine protease 2 (TMPRSS2) [1]. Therefore, it has been reported that COVID-19 has more severe effects on tissues where the ACE-2 receptor and TMPRSS2 enzyme are overexpressed [1]. The overexpression of these two proteins in endothelial cells may be the cause of extensive endothelial dysfunction due to COVID-19 [4]. The high blood supply to the genitourinary tissues indicates that these tissues are rich in endothelial cells, and therefore they are potential targets for SARS-CoV-2. When the literature is examined, there are some publications indicating that COVID-19 causes damage to various genitourinary tissues, including testicular, penile, and kidney tissues [4,5,6].

Erectile dysfunction (ED) is defined as the inability to achieve or maintain an erection sufficient for sexual intercourse. The etiological origin of ED can be organic, psychogenic, or mixed. Organic causes can be of vasculogenic, neuronal, or hormonal origin, while psychogenic causes include generalized anxiety disorder and major depressive disorder. One of the reasons for sexual problems during the pandemic may be the fear of couples who have to live together of infecting each other. However, regardless of its etiology, ED is known to result in the loss of self-confidence, decrease quality of life, and lead to relationship problems. In our clinical practice, a significant portion of patients that have presented to our outpatient clinic with ED since the beginning of the current pandemic report that their complaints started after a COVID-19 infection. When we reviewed the literature, we found some publications suggesting that there may be a relationship between COVID-19 and ED [4,5,6,7,8]. Some of these publications aimed to elucidate the etiology of post-COVID-19 ED [4,5,6,8]. However, to the best of our knowledge, no study has demonstrated whether ED that develops after COVID-19 is temporary or permanent based on a long-term evaluation.

This study aimed to evaluate whether there was an increase in the frequency of ED among individuals with a history of COVID-19, and, if there was, determine whether this situation improved over time.

## 2. Materials and Methods

This study was initiated after obtaining approval from the Clinical Research Ethics Committee of Health Sciences University Diskapi Yıldırım Beyazıt Training and Research Hospital affiliated with the Republic of Turkey Ministry of Health (approval date: 13 December 2021, number: 126/22). A total of 125 healthy male healthcare workers, 95 with and 30 without a history of COVID-19, were evaluated in terms of erectile function. The inclusion criteria were being aged 30–50 years, having been treated for COVID-19, being married or in a regular sexual relationship, and having had a regular income since the beginning of the pandemic. Excluded from the study were individuals who had received COVID-19 treatment in a hospital or intensive care unit, those with a history of cardiopulmonary involvement, those using phosphodiesterase-5 inhibitors/androgen replacement drugs/5-alpha-reductase inhibitors, and those with a diagnosis of psychiatric disease/diabetes mellitus/hypertension/erectile dysfunction. In order to evaluate the erectile function of the participants, they were asked to complete the five-item International Index of Erectile Function (IIEF-5). The total score in this questionnaire varies between 5 and 25 and classified as follows: 22–25, no ED; 17–21, mild ED, 12–16; mild to moderate ED; 8–11, moderate ED, and 5–7, severe ED. Four study groups were formed. The first three groups consisted of individuals who had a history of COVID-19 confirmed by the polymerase chain reaction (PCR) test at different times and recovered from the disease. Group 4 constituted the control group without a history of COVID-19 diagnosis. The details of the study groups are given below.

Group 1 (*n* = 30): COVID-19 PCR positivity within the past 6 months;
Group 2 (*n* = 31): COVID-19 PCR positivity 6 to 12 months before the study period;
Group 3 (*n* = 34): COVID-19 PCR positivity longer 12 months before the study period;
Group 4 (*n* = 30): No COVID-19 history.

### Statistical Analysis

G*Power v. 3.1.9.6 (Franz Faul, Universität Kiel, Kiel, Germany) package program was used to calculate the sample size. Considering an effect size of 0.40 according to one-way analysis of variance (ANOVA), it was determined that at least 96 individuals (at least 24 for each group) should be included in the study to test the statistical significance of differences between the groups at 90% power and 5% error level.

Data analysis was performed using IBM SPSS Statistics version 25.0 (IBM Corporation, Armonk, NY, USA). The assumptions of the normality of data distribution and variance homogeneity were checked using the Kolmogorov–Smirnov and Levene tests, respectively. Data were expressed as mean ± standard deviation or median (25th–75th percentile) values, where applicable. The mean differences in age between the groups were analyzed using one-way ANOVA, and the Kruskal–Wallis test was applied for the comparison of the IIEF-5 scores. When the *p*-value of the Kruskal–Wallis test was statistically significant, the Dunn–Bonferroni test was used to determine the group(s) that caused the significant difference. A *p* value of less than 0.05 was considered statistically significant.

## 3. Results

There was no statistically significant difference between the groups in terms of demographic and clinical data (*p* > 0.05) (Table 1).

**Figure 1:**
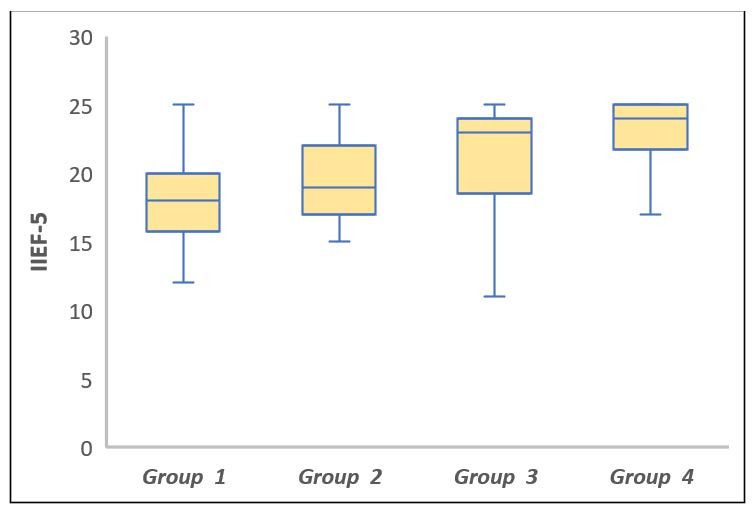
Comparison of the IIEF-5 scores between the study groups. The horizontal lines in the middle of each box show the median values, while the top and bottom borders of the box mark the 25th and 75th percentiles, respectively. The whiskers above and below each box indicate the maximum and minimum IIEF-5 scores.

A statistically significant difference was found between the groups in terms of the IIEF-5 scores (*p* < 0.001), and this difference was determined to be caused by the significantly higher IIEF-5 scores of Groups 3 and 4 compared Group 1 (*p* = 0.004 and *p* < 0.001, respectively). In addition, the IIEF-5 score of Group 4 was statistically significantly higher than that of Group 2 (*p* < 0.001). However, the IIEF-5 scores did not statistically significantly differ between Groups 1 and 2, Groups 2 and 3, and Groups 3 and 4 (*p* > 0.999, *p* = 0.204, and *p* = 0.592, respectively) (Table 2). Figure 1 presents the box plots of the IIEF-5 scores by study group.

## 4. Discussion

Although COVID-19 was considered to be limited to the respiratory system when it first emerged, it has, over time, been understood that this disease can also cause systemic involvement in many tissues and organs, such as the heart, kidneys, and vascular system in the period following the active infection [3,9]. The ACE-2 receptor, which is used by SARS-CoV-2 as a mediator to infect host cells, is highly expressed in the vascular endothelium [9]. Therefore, the virus has high affinity for the vascular endothelium and can lead to endothelial dysfunction [9]. As a result, vascular integrity is impaired. The integrity of penile cavernosal endothelial structures and the provision of blood flow are the basic requirements of erectile function, which also explains the effect of the virus on the development of ED.

Considering the increase in the number of men that presented to our outpatient clinic with the complaint of new-onset ED after COVID-19 during the pandemic period, we reviewed the literature concerning the relationship between these two conditions and found studies suggesting that there may be an increase in the frequency of ED after COVID-19 in parallel with our observations [3,7]. Chu et al. evaluated a database of 66 million patient records in the United States to determine the risk of post-COVID-19 ED [3]. In that study, men aged ≥ 18 years diagnosed with COVID-19 were compared with those without a diagnosis of COVID-19 during the same period [3]. The authors attempted to eliminate the effect of confounding variables using propensity score matching for demographics and comorbid medical conditions. After propensity score matching, they compared 230,517 men with COVID-19 to 232,645 men without COVID-19 and found that a COVID-19 diagnosis was significantly associated with ED (odds ratio: 1.20, *p* = 0.04) [3]. In another study with a smaller sample size examining the relationship between COVID-19 and ED development, Sansone et al. evaluated 25 patients with and 75 patients without a history of COVID-19 positivity in terms of ED [7]. They reported that the prevalence of ED was higher in patients with a history of COVID-19 than in those without this history (28% vs. 9.33%; *p* = 0.027).

In the literature, in addition to studies investigating whether there is an increase in the frequency of ED after COVID-19, there are also those exploring possible reasons for this increase [4,5,6,8]. Kresch et al. obtained penile tissue samples during penile prosthesis surgery from men with and without a history of COVID-19 to describe histopathological changes that could be caused by COVID-19 in penile tissue [4]. Using the electron microscopic examination, the researchers were able to demonstrate the presence of extracellular viral particles [peplomers (spikes)] around the penile vascular endothelium in patients with a history of COVID-19 but not in those without a history of COVID-19 [4]. When the same samples were further examined in terms of endothelial nitric oxide synthase, which indicates endothelial function, it was determined that the men with a history of COVID-19 had lower levels than those without a COVID-19 history. In the same study, the authors noted the presence of viral RNA with the PCR test in the specimens of patients with a history of COVID-19 [4]. There are also other publications on testicular damage in patients with a diagnosis of COVID-19 [6,10]. Pan et al. stated that 19% of patients diagnosed with COVID-19 experienced scrotal discomfort [10]. Yang et al. examined changes in the postmortem testicular tissues of men who died due to COVID-19 and found significant damage to the seminiferous tubules, mild lymphocytic inflammation, and a significant decrease in the number of Leydig cells [6]. When Leydig cells in the interstitium were counted in ten 400*microscopy fields, the mean number of Leydig cells per tubule cross-section was calculated as 2.2 (range: 0.44–5.3) in COVID-19 testes, which was significantly lower compared to the control group (7.8, range: 5.3–10, *p* < 0.001) [6]. The decrease in Leydig cells, which are responsible for most of the endogenous androgen production, in patients with COVID-19 may be one of the mechanisms that can explain post-COVID-19 ED. Cardiopulmonary disease caused by COVID-19 may also be one of the etiological causes of ED development, because good oxygenation is one of the basic requirements for good male sexual health. The decrease in systemic oxygenation due to pulmonary distress caused by COVID-19 causes a decrease in cavernous tissue oxygenation, which may be another factor that can lead to temporary erectile dysfunction [11].

In another study, Sevim et al. prospectively evaluated the effects of anxiety and depression on erectile function after COVID-19 [8]. The researchers observed that the erectile function scores (IIEF score, sexual desire, orgasmic function, intercourse satisfaction, and frequency of sexual intercourse) of the patients decreased in the post-COVID-19 period compared to the pre-COVID-19 period (*p* < 0.01). In addition, there was an increase in anxiety and depression scores (Generalized Anxiety Disorder 7 and Beck Depression Inventory) in the post-COVID-19 period compared to the pre-COVID-19 period (*p* < 0.01). The authors concluded that this deteriorated post-COVID-19 psychological state had a negative effect on erectile function and should not be overlooked [8]. In brief, ED may develop after COVID-19 as a result of certain organic or psychogenic factors. In our study, in which we evaluated whether this situation was permanent, we determined that the deterioration in erectile function that emerged with COVID-19 infection started to improve over time, especially after one year of the disease. We consider that this study is important since it is, to the best of our knowledge, the first to evaluate changes in erectile function after COVID-19 in the long term.

## 5. Conclusions

There may be deterioration in erectile function after COVID-19; however, this deterioration tends to improve over time, especially from the first year after active infection. Given that vascular, hormonal, and/or psychogenic factors may lead to the development of ED after COVID-19, we consider that in order to easily manage this process, it is important to determine the underlying cause, initiate appropriate treatment, and inform couples that this situation may be temporary.

## Figures and Tables

**Table 1:** Comparison of the study groups in terms of demographic and clinical characteristics.

	Age	BMI	Smoking	Alcohol Consumption
Group 1	40.1 ± 5.6	26.94 ± 4.07	11/30 (36.6%)	4/30 (13.3%)
Group 2	40.3 ± 6.5	27.03 ± 4.18	12/31 (38.7%)	4/31 (12.9%)
Group 3	41.5 ± 7.1	26.04 ± 4.03	12/34 (35.2%)	5/34 (14.7%)
Group 4	40.2 ± 6.5	27.13 ± 4.25	10/30 (33.3%)	3/30 (10%)
*p*-Value	0.793	0.904	0.814	0.384

BMI: body mass index, Group 1: <6 months since COVID-19 positivity, Group 2: 6–12 months since COVID-19 positivity, Group 3: >12 months since COVID-19 positivity, Group 4: no COVID-19 history.

**Table 2:** Comparison of the study groups in terms of the IIEF-5 scores.

IIEF-5 Scores	
Group 1	18 (16–20) ^a,b^
Group 2	19 (17–22) ^c^
Group 3	23 (18–24) ^a^
Group 4	24 (22–25) ^b,c^
*p*-Value	<0.001 ^‡^

^‡^ Kruskal–Wallis test. ^a^ Group 1 vs. 3 (*p* = 0.004), ^b^ Group 1 vs. 4 (*p* < 0.001), ^c^ Group 2 vs. 4 (*p* < 0.001). IIEF: International Index of Erectile Function, Group 1: <6 months since COVID-19 positivity, Group 2: 6–12 months since COVID-19 positivity, Group 3: >12 months since COVID-19 positivity, Group 4: no COVID-19 history.

## Data Availability

Data cannot be shared for ethical/privacy reasons.
